# Hedgehog Inhibitor and Paclitaxel: A Phase I Trial of the Oral Hedgehog inhibitor Taladegib in Combination with Weekly Paclitaxel in Patients with Advanced Solid Tumours

**DOI:** 10.1007/s10637-026-01612-4

**Published:** 2026-05-02

**Authors:** Rosalind Glasspool, Sarah Patricia Blagden, Michelle Lockley, Elaine McCartney, Carol Evans, Fiona Thomson, Jennifer Brown, Laura Spiers, Chantevy Pou, Liz-Anne Lewsley, Iain A. McNeish, T.R. Jeffry Evans

**Affiliations:** 1 Beatson West of Scotland Cancer Centre and School of Cancer Sciences, University of Glasgow, Glasgow, UK; 2 Early Phase Clinical Trials Unit, Churchill Hospital, Oxford University Hospitals, Oxford, OX3 7LE UK; 3 Centre for Cancer Centre for Cancer Evolution, Barts Cancer Institute, Queen Mary University of London, London, UK; 4 Cancer Division, University College London Hospital, London, UK; 5 Glasgow Oncology Clinical Trials Unit, University of Glasgow, Glasgow, UK; 6 The Translational Pharmacology & Immunology Laboratory, School of Cancer Sciences, University of Glasgow, Glasgow, UK; 7 Ovarian Cancer Action Research Centre, Department of Surgery and Cancer, Imperial College London, London, UK

**Keywords:** Hedgehog inhibitor, Taladegib, Paclitaxel, Phase I trial

## Abstract

The hedgehog (Hh) signalling pathway is aberrantly activated in solid tumours. Inhibition of Hh signalling by SMO inhibitors is effective in the treatment of basal cell carcinomas and medulloblastomas whereas combination treatments may be required in other solid tumours. When given with an SMO inhibitor in preclinical models, the efficacy of paclitaxel is enhanced and there is potential reversal of mechanisms of resistance to paclitaxel supporting clinical investigation of the combination. In this phase I trial, the safety and feasibility of the oral SMO inhibitor, taladegib, was evaluated in combination with weekly paclitaxel in patients with advanced solid tumours. This was an open-label, nonrandomised, multicentre, dose escalation trial using a standard 3 + 3 design (EudraCT: 2014-004695-37 ISRCTN15903698). Primary objectives were to determine dose-limiting toxicities and the maximum tolerated dose of the combination. Patients received up to 6 cycles of paclitaxel 80 mg/m2 (day 1, 8 and 15 of a 28-day cycle). Then, 16 patients were recruited into 3 cohorts and received taladegib at 100 mg, 200 mg and 400 mg once daily, respectively. Grade 2/3 peripheral sensory neuropathy was the dose-limiting toxicity. The maximum tolerated dose of taladegib in combination with weekly paclitaxel was 200 mg once daily. Four patients had a partial response and 4 had confirmed stable disease at 16 weeks. The combination is feasible but cumulative neuropathy may limit longer term treatment. Hh signalling may be implicated in chemotherapy-induced neuropathy.

**Supplementary Information:**

The online version contains supplementary material available at 10.1007/s10637-026-01612-4.

## Introduction

The Hedgehog (Hh) signalling pathway plays an essential role in early development, and its aberrant activation is implicated in carcinogenesis and tumour growth [1]. The pathway consists of three secreted ligands: Sonic Hedgehog (SHh), Indian Hedgehog (IHh) and Desert Hedgehog (DHh). These bind to the negative regulatory membrane receptor, patched (PTCH). PTCH normally inhibits the membrane protein smoothened (SMO). Binding of an Hh ligand abrogates the negative effect of PTCH on SMO. Activated SMO results in activation and release of Glioma-associated oncogene (GLI) transcription factors (GLI-1, GLI-2 and GLI-3) which translocate to the nucleus and induce expression of multiple genes involved in differentiation, proliferation and survival.

Germline and somatic mutations of PTCH are associated with basal cell carcinomas (BCC) and medulloblastomas and inhibition of Hh signalling by small-molecule inhibitors of SMO induces clinical responses in these tumours [2, 3]. Ligand-dependent activation has been implicated in other solid tumours including breast, lung, prostate, pancreatic cancers [4] and in ovarian carcinomas where it appears to be associated with poor prognosis and higher expression is found in recurrent or persistent tumours [5, 6]. Inhibition of Hh signalling results in reduced growth and invasiveness in tubo-ovarian cell line models and a sustained response to platinum-taxane chemotherapy in xenograft models [7, 8]. Paclitaxel is used in the treatment of several solid tumours and the weekly schedule is well tolerated, making it an appropriate chemotherapy backbone for combination with novel therapies. However, despite activity in a number of tumours, resistance is a major clinical problem. For example, response rates in platinum-resistant ovarian cancer are between 22 and 55% but responses are short-lived with median progression-free survival of 3–5 months [9–11].There is preclinical evidence that several potential mechanisms of taxane resistance can be reversed by the addition of Hh inhibitors including reversal of an increase in stem-like cells, an increase in micro RNS 200c and decreased expression of MDR1 [12–14].

Taladegib is a potent oral inhibitor of SMO. It has been shown to be safe and tolerable at a recommended monotherapy dose of 400 mg, once daily. It has biologically effective pharmacodynamic effects at doses of ≥ 50 mg/day with objective responses in basal cell carcinomas [15].

In this trial, we investigated the safety and feasibility of the combination of weekly paclitaxel and continuous oral taladegib in patients with advanced solid tumours. It was originally designed as a phase I/Ib trial with an expansion phase in women with platinum-resistant epithelial ovarian cancer. However, phase Ib was not performed, as after the sale of taladegib to another company, a supply with sufficient shelf life for the duration of the expansion cohort was not available. We considered it unethical to commence the expansion cohort without confirmed supply for all patients for the expected duration of the trial. So, we report here the Ph1 dose escalation part of the study.

## Methods

This was an open-label, nonrandomised, multi-centre, dose escalation trial using a standard 3 + 3 design. Patient eligibility included histologically or cytologically confirmed advanced solid tumours refractory to standard therapy or for whom weekly paclitaxel was considered to be an appropriate therapy; ECOG performance status of 0–2, life expectancy of at least 3 months, no history of ≥ grade 2 peripheral neuropathy during prior treatment and no > grade 1 peripheral neuropathy at the time of study entry (see supplementary data for full eligibility criteria).

Patients received up to 6 cycles of paclitaxel 80 mg/m^2^ given by intravenous infusion over 1 h on day 1, 8 and 15 of a 28-day cycle alongside once-daily oral taladegib at 100 mg, 200 mg and 400 mg, depending on dose cohort. The starting dose of 100 mg, 2 dose levels below the single-agent maximally tolerated dose, was selected due to the potential for overlapping toxicities of fatigue, dysgeusia, anorexia, nausea and vomiting, diarrhoea, alopecia, rash, myalgia and asthenia. There was also the potential for an interaction resulting in an increase in paclitaxel levels as a result of inhibition of CYP2C8 by taladegib and its active metabolite LSN3185556. In addition, at least 50% inhibition of GLI-1 was seen in skin biopsies in the single-agent phase I trial at the lowest dose of 50 mg of taladegib suggesting that 100 mg could be an effective dose. The starting dose of paclitaxel was not reduced to avoid compromising the clinical efficacy of the paclitaxel.

The primary objectives were to determine dose-limiting toxicities (DLTs) and maximum tolerated dose (MTD) of the combination. Secondary objectives were safety and tolerability; the recommended phase II dose (RP2D) and the pharmacokinetics of taladegib and paclitaxel when given in combination. Exploratory objectives in the dose escalation phase were to investigate the expression of GLI-1; the frequency and pattern of expression of the Hh pathway proteins and the frequency of mutations in Hh pathway genes in archival samples and the relationship between total cfDNA in serial samples and response to treatment.

DLTs were based on clinical and laboratory toxicity assessments (NCI-CTC version 4.03) occurring during the 1st cycle and considered related to treatment with either drug. DLTs were defined as any of the following: failure to deliver > 75% of planned taladegib doses due to toxicity, omission of > 1 dose of paclitaxel due to toxicity; ≥ Grade 3 neutropenia with sepsis or fever > 38.5 °C; Grade 4 neutropenia lasting > 7 days; Grade 4 thrombocytopenia (of any duration); ≥ Grade 3 Nausea and vomiting not controlled by maximal antiemetics; Grade 3 hyponatraemia that did not resolve in ≤ 7 days or grade 4 hyponatraemia; any other ≥ Grade 3 toxicity; and ≥ Grade 2 peripheral neuropathy. Patients who did not receive > 75% of taladegib and at least 2 doses of paclitaxel during the first cycle, for reasons other than toxicity, were not evaluable for DLT and were replaced. If no DLTs occur in the first three patients, the dose was escalated to the next dose level. If one patient experienced a DLT the cohort was expanded to up to 6 patients. If 2 or more of 3–6 patients experienced a DLT, no more patients were treated at that dose level and the dose level below was expanded to 6 patients if this had not already occurred and if < 2 patients experience DLT, this was declared the maximum tolerated dose level.

The study was approved by the West of Scotland Research Ethics Committee (EudraCT: 2014–004695-37 ISRCTN15903698). All participants gave written informed consent prior to undergoing any study-related procedures.

### Pharmacokinetic sampling schedule

Taladegib was started on day 2 of cycle 1. Both taladegib and its active metabolite, LSN3185556, were measured. Blood samples were taken at the following times:

Paclitaxel alone: cycle 1 day 1: preinfusion, 1, 2, 4 and 6 h after start of infusion.

Taladegib and paclitaxel in combination: cycle 1 day 15: pre-infusion, 1, 2, 4 and 6 h after the start of infusion.

Taladegib alone: cycle 1 day 22: pre-taladegib, 3 and 6 h postdose.

Blood for germline DNA was collected at baseline and plasma samples for cfDNA were taken on day 1 of cycles 1–6, at the end-of-treatment visit and at progression.

### Statistical analysis

All analyses were descriptive and were conducted using SAS v9.4. All eligible patients who received at least two cycles of treatment were assigned a response category. Responses were defined by RECIST v1.1 for all patients.

### Translational biomarker analysis

#### Materials

For PK samples, 5 ml of blood was taken into EDTA tubes and gently inverted 8–10 times. Within 60 min, it was centrifuged at 1500–2000 g for 20 min at room temperature. Plasma supernatant was transferred into 2 × 2 ml Eppendorf tubes and stored at − 80 °C. For PK analysis, certified standards were paclitaxel (Sigma-Aldrich), docetaxel (Fluka) and taladegib (LY2940680 and LSN2940680 (LSN3185556 HCl), Eli Lilly, Indianapolis, IN, USA)**.** For RNAscope ISH studies, all reagents and probes were from ACD, BioTechne as follows: dihydrodipicolinate reductase (dapB) (RNAscope® 2.5 LS Duplex negative control probe), Hs-PPIB (RNAscope® 2.5 LS duplex control probe, PPIB-C1), Hs-Polr2a (RNAscope® 2.5 LS duplex control probe, Polr2a-C2), Hs-SMO and Hs-Gli1 in combination with RNAscope 2.5 LS reagent kit. Tumour DNA and RNA extractions were conducted using the AllPrep DNA/RNA FFPE kit, and plasma-cell-free DNA was isolated using the QIAamp circulating nucleic acid kit (QIAGEN Ltd, Manchester, UK). The nCounter® Pan-Cancer panel was obtained from Nanostring (Seattle, WA, USA). The Pan-Cancer (524) TMB/MSI targeted sequencing panel was from Nonacus (Birmingham, UK). The Qubit dsDNA HS assay kit was from Thermo Fisher (Loughborough, UK).

For germline DNA analysis, blood was collected into 6-ml EDTA tubes, which were gently inverted 6–7 times to allow blood to mix with the tube additive, and stored at − 80 °C.

For ctDNA analysis, 8–9 ml of blood was collected into an EDTA tube and gently invert tubes 8–10 times. Within 1 h after collection, it was centrifuged at 1600 g for 10 min and 1.5-ml aliquots of the upper plasma layer transferred to sterile 2-ml nonscrew capped RNase-free microtubes. Plasma aliquots were centrifuged at 14,000 rpm for 10 min. Then, 1.5-ml aliquots of supernatant were transferred to 2-ml screw-capped microtubes, and stored at − 80 °C.

### Methods

#### Drug pharmacokinetic analysis

Paclitaxel levels in plasma were measured using a sensitive high-performance liquid chromatography (HPLC) assay with a Shimadzu Prominence Quaternary HPLC system and associated LabSolutions software. Analysis of taladegib and its active metabolite, LSN3185556, in plasma was conducted by Charles River Laboratories (Montreal, Canada) using a liquid chromatography-tandem mass spectrometry LC/MS/MS assay.

### RNA in situ hybridisation (RNA-ISH) analysis usingRNAScope

For the single-plex and duplex automated RNAscope assays [16], 4-µm tissue sections were processed following manufacturer’s instructions and were baked and deparaffinized on the Leica Biosystems BOND RX platform, followed by target retrieval and protease treatment (15 min at 40 °C). Probes were then hybridized for 2 h at 42 °C, followed by RNAscope amplification. For the LS automated single-plex assay, DAB chromogenic detection was then performed. For the LS automated duplex RNAscope assay, fast red chromogenic detection was performed first, followed by DAB chromogenic detection.

### Gene expression analysis

Marked sections of FFPE tumour tissues (10 µm) on slides were excised and deparaffinized using a standard xylene/ethanol method, and DNA and RNA were extracted. Multiplex gene expression analysis was performed on extracted RNA using Nanostring’s nCounter® Pan-Cancer Pathways panel following manufacturer’s instructions. Data were analysed by ROSALIND® (https://rosalind.onramp.bio/), with a HyperScale architecture developed by OnRamp BioInformatics, Inc. (San Diego, CA).

### Targeted sequencing of tumour DNA

Targeted sequencing of DNA extracted from tumour FFPE samples was performed using Nonacus’ Pan-Cancer (524) TMB/MSI panel from Nonacus, according to the manufacturer’s guidelines.

#### Isolation and measurement of cell-freeDNA (cfDNA) from plasma

cfDNA was isolated from plasma using the QIAamp circulating nucleic acid kit following the manufacturer’s directions. The isolated cfDNA concentration was determined using the QUBIT dsDNA HS assay kit following the manufacturer’s instructions.

## Results

A total of 16 patients were included from 3 centres in the UK: 3 in dose level 1 (100 mg), 6 in dose level 2 (200 mg) and 7 in dose level 3 (400 mg). One patient in level 3 was not evaluable for DLT and was replaced. Median age was 61.5 years (range 34–79) (see supplementary table S1 for baseline characteristics).

There were no DLTs in cohort 1 or the first 3 patients in cohort 2. In cohort 3, 1 of 3 patients developed grade 2 peripheral sensory neuropathy on day 21 of cycle 1. The cohort was therefore expanded. Then, 2 further patients developed grade 2 peripheral neuropathy during cycle 1. Sensory peripheral neuropathy was defined as the DLT. Cohort 2 was expanded to 6 patients with no further DLTs. A dose of 200 mg of taladegib once daily was declared the MTD in combination with weekly paclitaxel 80 mg/m^2^ on day 1, 8 and 15 of a 28-day cycle. Four further patients experienced ≥ grade 2 neuropathy in later cycles, 2 in cohort 1 at cycle 6 and 2 in cohort 2 at cycle 2 and cycle 5. Grade 2 sensory neuropathy is defined as moderate symptoms affecting instrumental activities of daily living and hence has potential for significant impact on patient quality of life.

Cumulative (all cycles) grade-3/4 toxicities (worst grade per patient) are shown in Table 1. Table 2A shows the number of cycles of paclitaxel completed per patient in each dose level and the number of dose reductions. In total, 5 patients completed the full six cycles of weekly paclitaxel and 11 patients discontinued before completing 6 cycles: 6 due to progressive disease, 1 due to treatment-related toxicity and 4 for other reasons. The most common reasons for dose reductions of paclitaxel were peripheral neuropathy (8) and neutropenia (2).

**Table 1:** Cumulative (all cycles) Drug- Related Grade 3/4 toxicity (worst grade per patient)

		**Dose level 1N (%)Total in DL1=3**	**Dose level 2N (%)Total in DL2=6**	**Dose level 3N (%)Total in DL3=7**	**All N (%)Total in study= 16**
**Anaemia**	**Grade 3**	0 (0)	0 (0)	1 (14.3)	1 (6.3)
**WBC decrease**	**Grade 3**	1 (33.3)	1 (16.7)	0 (0)	2 (12.5)
**Lymphocyte count decrease**	**Grade 3**	0 (0)	1 (16.7)	1 (14.3)	2 (12.5)
**Neutrophil count decreased**	**Grade 3**	0 (0)	1 (16.7)	0 (0)	1 (6.3)
	**Grade 4**	1 (33.3)	0 (0)	0 (0)	1 (6.3)
**Peripheral sensory neuropathy**	**Grade 3**	0 (0)	0 (0)	1 (14.3)	1 (6.3)
**Musculoskeletal and connective tissue disorder **	**Grade 3**	0 (0)	0 (0)	1 (14.3)	1 (6.3)
**Fatigue**	**Grade 3**	0 (0)	0 (0)	2 (28.6)	2 (12.5)
**GGT increased**	**Grade 3**	1 (33.3)	0 (0)	0 (0)	1 (6.3)
**Hyperglycaemia**	**Grade 3**	0 (0.0)	0 (0)	1 (14.3)	1 (6.3)
**Hypokalaemia**	**Grade 3**	1 (33.3)	0 (0)	0 (0)	1 (6.3)

**Table 2:**
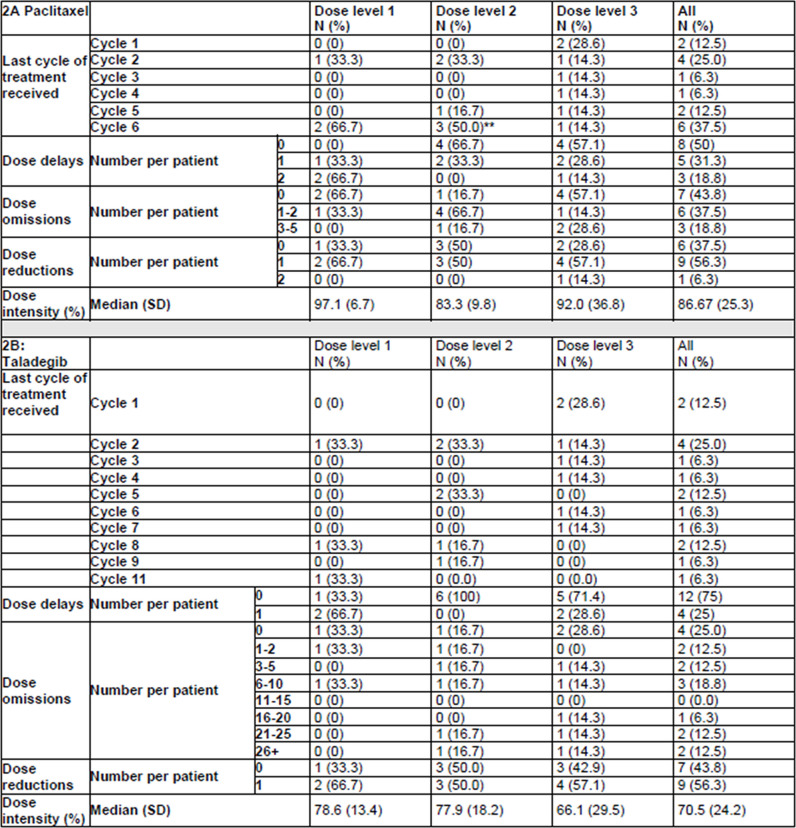
Treatment compliance

The median dose intensity of paclitaxel for cohorts 1, 2 and 3 was 97%, 83% and 92%, respectively. Reasons for stopping treatment are shown in Table S2. Table 2B shows the number of cycles of taladegib started per patient and the number of missed doses and dose reductions. The most common reasons for missed doses were patient error (7 patients) and toxicity (8 patients). The most common toxicities resulting in missed doses were nausea, vomiting and muscle cramps. Nine patients required one dose reduction of taladegib, for peripheral neuropathy (6), neutropenia (5) and muscle cramps (2). The median dose intensity of taladegib for dose levels 1, 2 and 3 was 79% 78% and 66%.

**Fig. 1:**
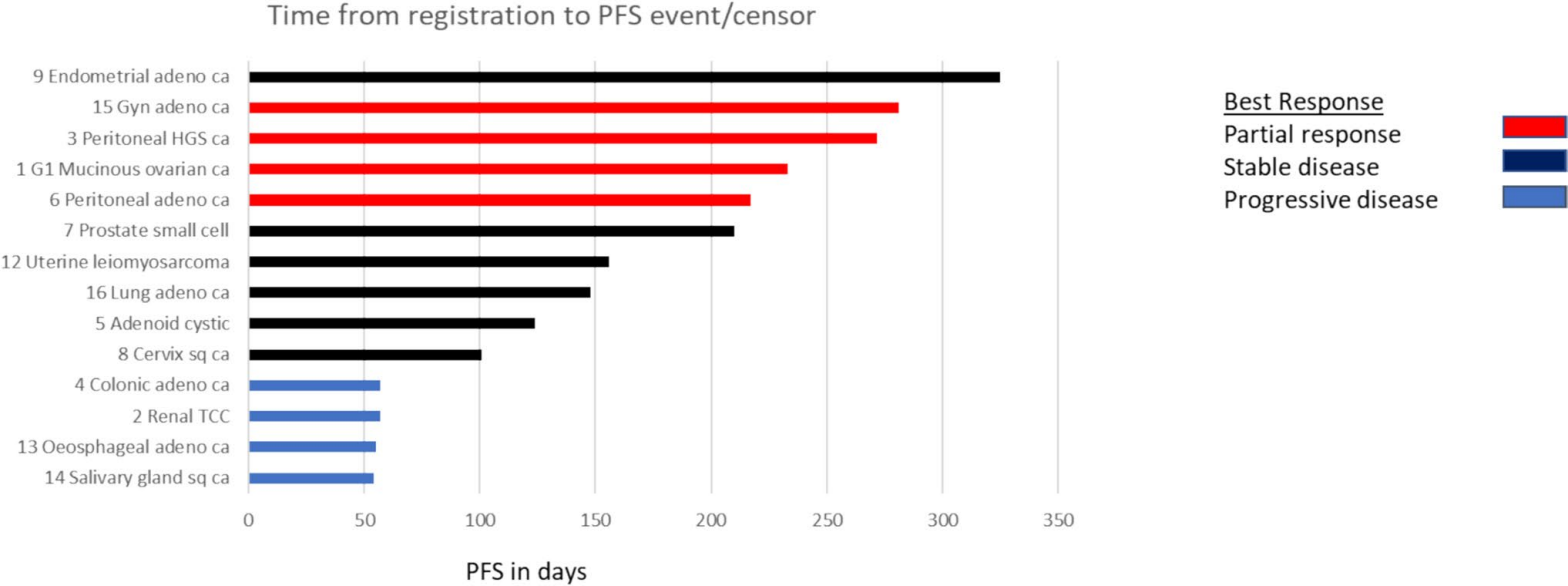
Time from registration to progression event or censoring in days

Four patients had a partial response (low-grade mucinous ovarian carcinoma, high-grade serous peritoneal carcinoma, peritoneal adenocarcinoma and an adenocarcinoma of the female genital tract). Six patients had a best response of stable disease with four having confirmed stable disease for at least 16 weeks (endometrial carcinoma, adenocarcinoma of the lung, high-grade leiomyosarcoma of the uterus and small-cell carcinoma of the prostate) (Fig. 1).

### Pharmacokinetics

**Fig. 2:**
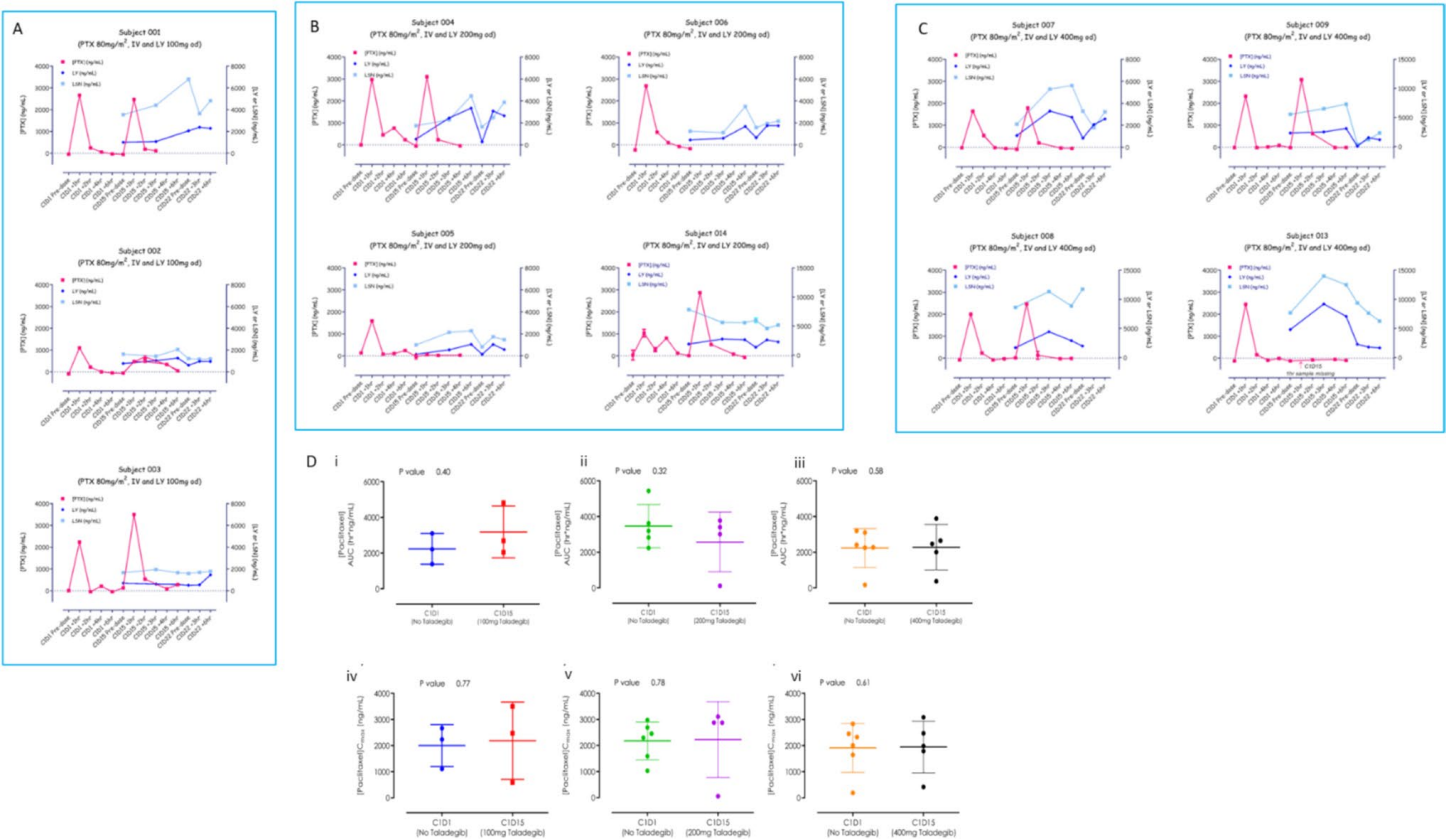
PK analysis of paclitaxel (pink), taladegib (dark blue) and LSN3185556 (light blue) for individual patients with samples available. (**A**) Dose level 1, taladegib 100 mg. (**B**). Dose level 2 taladegib 200 mg. (**C**) Dose level 3 taladegib 400 mg. (**D**). Paclitaxel area under the concentration-time curve (AUC) observed (mean +/−- 1 standard deviation) with paclitaxel alone (before C1D1) and with paclitaxel and taladegib (C1D15) at dose level: 100 mg (i), 200 mg (ii) and 400 mg (iii). Paclitaxel maximum plasma concentration (Cmax) observed with paclitaxel alone (before C1D1) and paclitaxel and taladegib (C1D15) at dose level 100 mg, (iv) 200 mg (v) and 400 mg (vi).

PK samples were available for 11/16 patients. Statistical analysis was not performed in this small sample size; however, the maximum concentration and area under the curve (AUC) of paclitaxel did not show an obvious difference between cycle 1, day 1 (paclitaxel alone) and cycle 1, day 15 (paclitaxel/taladegib combination) at any dose level in this limited sampling schedule (Fig. 2D). Administration of paclitaxel on day 15 did not appear to affect the concentration of taladegib or its main metabolite, LSN3185556 on day 15 (combination) compared to day 21 when taladegib was given alone (Fig. 2A–2C). Nor was there suggestion of accumulation of paclitaxel, taladegib or LSN3185556 over the sampling period.

### Hedgehog pathway biomarker analysis in tumour samples

**Fig. 3:**
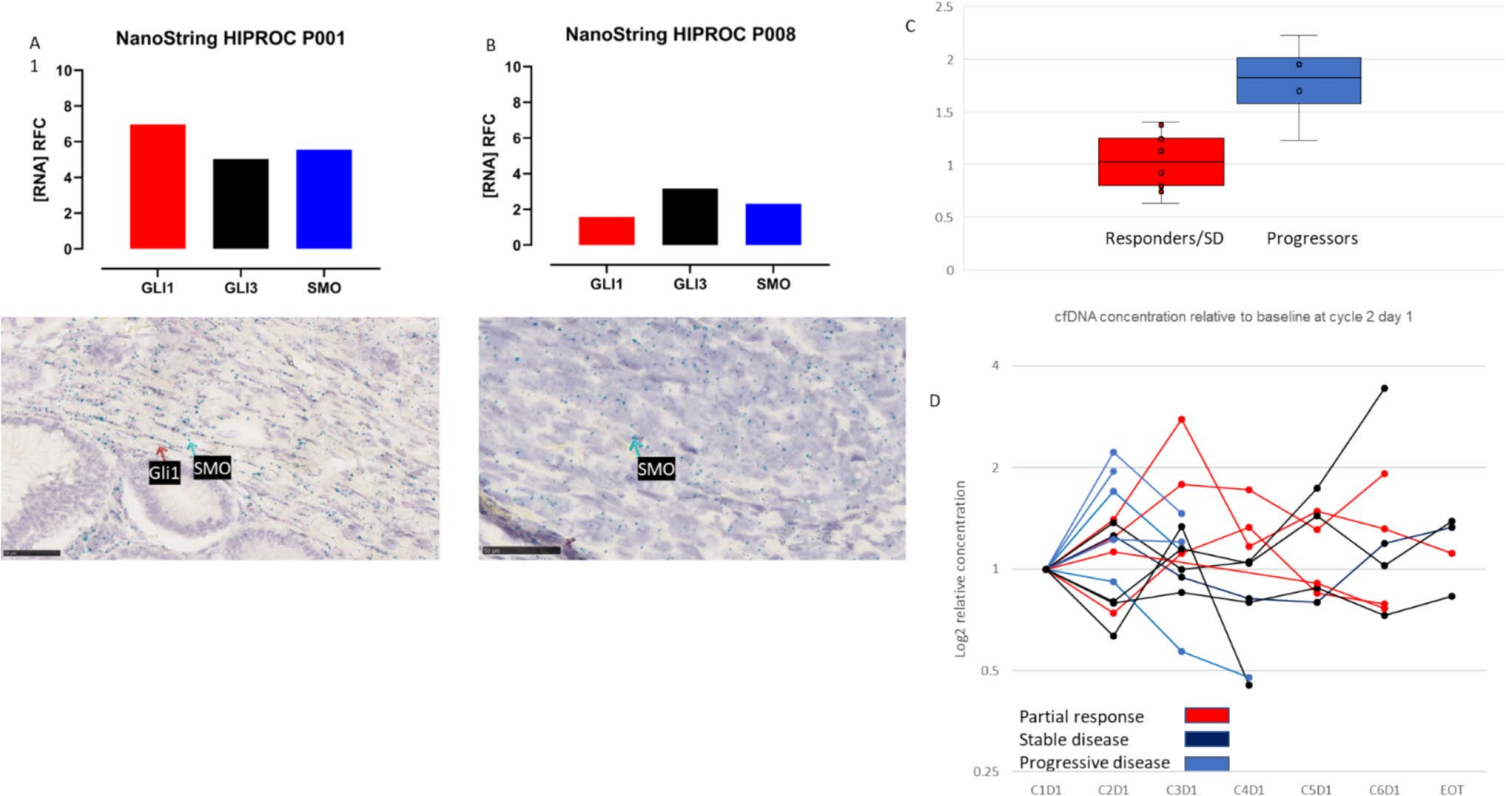
Representative examples of (**A**) high and (**B**) low expressions of GLI and SMO by Nanostring and on RNA scope. A is from patient 1, low-grade ovarian mucinous carcinoma and B is from patient 8, squamous cell carcinoma of cervix. (**C**) Relative concentration of cfDNA on cycle 2 day 1 compared to baseline in patients with best response of partial response or stable disease compared to those with progressive disease. (**D**) cfDNA concentration relative to baseline over time (C1D1=cycle 1 day 1 etc. ; EOT = end of treatment.)

Archival tumour samples were available for 10 patients and used to explore whether aberrations in the Hedgehog pathway were present using in situ hybridisation analysis of *Gli* and *SMO* gene expression by RNAscope and mRNA expression of *GLI 1, 3, PTCH* and *Smo* by Nanostring. Representative examples are shown in Fig. 3A and 3B shows representative examples. A range of expression across samples was seen using both techniques, suggesting that they may offer a means of assessing Hh pathway activation, but their reliability, validity and clinical relevance will need to be investigated in larger cohorts. Assessment of the relationship between Hh pathway inhibition and response required a randomised design in the setting of a combination treatment and so was outside the scope of this study.

Since mutations in Hh pathway genes might confer sensitivity or resistance to Hh inhibitors, we performed targeted sequencing of tumour tissue using Nonacus panels. This identified pathogenic or likely pathogenic mutations in all samples, but no mutations in *PTCH* were found in this small cohort of mixed tumours. One patient had a mutation in *SUFU* (SCC of prostate). This patient had stable disease by RECIST 1.1, but since *SUFU* is downstream of SMO, it would be expected to confer resistance to taladegib.

Reliable early markers of response would allow patients to stop ineffective treatments sooner thus avoiding toxicity and reducing cost. We therefore analysed cell-free DNA (cfDNA) samples for total cfDNA over time to assess whether changes in total cfDNA could act as an early marker of response. Although there was an increase in cfDNA concentration relative to baseline at cycle 2 day 1 in those with a subsequent best response of progressive disease compared to those with stable disease or partial response (Fig. 3C), this difference was not maintained over subsequent sampling points (Fig. 3D).

## Discussion

Taladegib, an oral inhibitor of SMO, can be combined with standard doses of weekly paclitaxel. The maximum tolerated dose is 200 mg once daily with 80 mg/m^2^ paclitaxel given i.v. on day 1, 8 and 15 of a 28-day cycle, with the dose-limiting toxicity of peripheral neuropathy. The toxicity profile for the combination was similar to that expected for each drug as monotherapy. However, the severity and frequency of peripheral neuropathy were higher than expected. In this study, 7 out of 16 (44%) patients experienced at least Grade 2 neuropathy. In contrast, in a randomised trial of weekly paclitaxel with, or without, visusertib in women with platinum-resistant ovarian cancer which used the same dosing schedule and grading system, the incidence of Grade 2 neuropathy was less than 10% in each arm^11^. The neuropathy was cumulative, which may limit the feasibility of longer-term treatment.

Hedgehog signalling has a multifaceted role in peripheral nerve repair and homeostasis. Following peripheral nerve injury, Schwann cells undergo extensive reprogramming, to promote and guide axonal repair [17]. This involves the upregulation of several genes including SHH. The neuroprotective role of SHH is suggested by several studies of different peripheral nerve injury models [18–21]. SHH may be involved in promotion of axonal survival, clearing of myeline and axonal debris necessary for nerve regeneration, neovascularisation and regenerative track formation [22]. Furthermore, injections of the Hh inhibitor, cyclopanime in the vicinity of the sciatic or infra-orbital nerve in rats mimicked the changes seen following chronic constriction injuries. Whilst exogenous SHH treatment promoted neurite formation and improved erectile dysfunction after injury to the cavernous nerve in a rat prostatectomy model [23, 24]. Similarly, disruption of normal Hh function is implicated in the aetiology of diabetes-induced peripheral nerve dysfunction and delivery of exogenous Hh proteins may have therapeutic potential in diabetic neuropathy [25]. Since paclitaxel induces axonal degeneration and demyelination [26], we hypothesise that inhibition of the Hedgehog pathway may have impaired repair of paclitaxel induced peripheral neuropathy.

Defining the characteristics of an activated Hh pathway is challenging without effective, standardised antibodies for immunohistochemistry. Expression of *GLI1, GLI3* and *SMO* can be demonstrated by RNA ISH and Nanostring gene expression analysis. We found a range of expression suggesting these techniques may offer a means of assessing Hh pathway activation, but further studies are needed to quantify this and define what constitutes an activated pathway. A limitation of the study was that we used archival samples rather than samples taken immediately pretreatment so we could not investigate the state of activation at the time of treatment. Premature closure of the trial before the expansion cohort meant that we could not examine the effect of taladegib on GLI expression in matched tumour samples that were planned for this cohort. However, a previous study of taladegib in BCC demonstrated a significant reduction in GLI expression measured by RT-PCR in skin samples at doses of 50 mg and above and a study of neoadjuvant treatment with another SMO inhibitor, sonedagib, in prostate cancer demonstrated ≥ twofold reduction in GLI expression [27] indicating that significant pharmacodynamic effects can be achieved at clinically tolerable doses. However, whether this translates into a therapeutic effect has yet to be demonstrated.

Further investigation in larger sample sizes is required to assess whether an increase in cfDNA at day 1 of cycle 2 relative to baseline could be used as an early predictor of progression. Interestingly, a subsequent study showed that inhibition of GLI1 reduces expression of FANCD2 and results in homologous repair deficiency in ovarian cancer cell lines. The combination of a GLI1 inhibitor and PARP inhibitor was synergistic in ovarian cancer mouse xenograft models [28] suggesting that further investigation of combinations of other Hh inhibitors and standard therapy is warranted.

## Conclusion

 We demonstrated that the maximum tolerated dose of the combination of taladegib and weekly paclitaxel is 200 mg of taladegib, orally, once daily with intravenous paclitaxel 80 mg/m^2^ on days 1, 8 and 15 of a 28-day cycle. The DLT is sensory peripheral neuropathy. The combination is feasible but cumulative neuropathy may limit longer term treatment.

## Supplementary Information

Below is the link to the electronic supplementary material.

ESM 1 (DOCX 29.9 KB)

## Data Availability

The data are available upon request from the Glasgow Oncology Trials Unit.
