# Supplementary material for: Hedgehog Inhibitor and Paclitaxel: A Phase I Trial of the Oral Hedgehog inhibitor Taladegib in Combination with Weekly Paclitaxel in Patients with Advanced Solid Tumours
Source: Invest New Drugs. 2026 May 2;44(3):264–73. doi: 10.1007/s10637-026-01612-4 (PMC13486132; doi:10.1007/s10637-026-01612-4)
Supplement: Supplementary file 1 [file 10637_2026_1612_MOESM1_ESM.docx]

# Supplementary Data

## Eligibility Criteria

1. Histologically or cytologically confirmed advanced solid tumours refractory to standard therapy or for whom weekly paclitaxel is considered, by the investigator, to be an appropriate therapy.
2. Written informed consent
3. Performance status ≤2 (ECOG)
4. Estimated life expectancy ≥ 3 months
5. Age ≥ 18 years
6. Adequate haematological, renal and hepatic function as defined by:
   - Haemoglobin (Hb)≥ 10g/dl
   - Neutrophil Count≥ 1.5 x 109/l
   - Platelets≥ 100 x 109/l
   - INR <2 x ULN and prothrombin time and activated partial thromboplastin time < 1.5 x ULN in the absence of therapeutic anticoagulation
   - Glomerular Filtration rate of ≥50 mL/ min (calculated using the Wright formula (Appendix IV) or measured by EDTA clearance. If both are performed the EDTA clearance should be used).
   - Total bilirubin ≤1.5 x ULN and ALT and AST ≤ 2.5 x ULN
   - Sodium above the lower limit of the institutional normal range
7. No history of ≥grade 2 peripheral neuropathy at any time during prior treatment and no greater than grade 1 residual peripheral neuropathy.
8. No use within the last 7 days of or requirement to continue medications that are strong inhibitors of CYP3A4 (Appendix V).
9. No prior treatment with taladegib (LY2940860/RXDX-109) LY2940680 or other Hedgehog pathway inhibitor.
10. Female patients with reproductive potential (i.e. patients whose reproductive organs remain in place and who have not passed the menopause) must have a negative serum pregnancy test within 7 days of trial enrolment and agree to use two highly effective forms of contraception during and for 6 months after last dose of treatment. Male patients of childbearing potential and their female partner must also agree to use a highly effective form of contraception during and for 6 months after treatment. Men with pregnant or lactating partners should be advised to use barrier method contraception to prevent exposure to the foetus or neonate
11. No symptoms or signs of gastrointestinal obstruction requiring parenteral nutrition or hydration or any other gastro-intestinal disorders or abnormalities, including difficulty swallowing, that would interfere with drug absorption, including ileostomies.
12. Ability to swallow capsules.
13. No significant cardiovascular diseases, including uncontrolled hypertension, clinically relevant cardiac arrhythmia, unstable angina or myocardial infarction within 6 months prior to registration, congestive heart failure ≥NYHA III, severe peripheral vascular disease, clinically significant pericardial effusion.
14. A corrected QT interval (QTc) of ≤470 msec on screening electrocardiogram (ECG).
15. No treatment within 28 days prior to registration with any investigational drug, radiotherapy, immunotherapy, chemotherapy, hormonal therapy (excluding HRT and long term androgen blockade for prostate cancer) or biological therapy. Palliative radiotherapy may be permitted for symptomatic control of pain from bone metastases, provided that the radiotherapy does not affect target lesions.
16. No serious infections in particular if requiring systemic antibiotic (antimicrobial, antifungal) or antiviral therapy, including known hepatitis B and/or C infection and HIV-infection.
17. No symptomatic CNS metastasis or leptomeningeal carcinomatosis.
18. No other severe concurrent disease, which may increase the risk associated with trial participation or trial drug administration and, in the judgement of the investigator, would make the patient inappropriate for entry into this trial, including significant neurologic, psychiatric, infectious, hepatic, renal, or gastrointestinal diseases or laboratory abnormality.
19. No known, uncontrolled hypersensitivity to the investigational drugs or their excipients.
20. No psychological, familial, sociological or geographical consideration potentially hampering compliance with the trial protocol and follow up schedule.
21. No known SIADH or adrenal insufficiency.

## Table S1: Baseline Characteristics

|  | Cohort | | | Total |  |
| --- | --- | --- | --- | --- | --- |
|  | Dose level 1 100mg | Dose level 2  200mg | Dose level 3  300mg |  |  |
|  |  |  |  |  |  |
| Number | 3 | 6 | 7 | 16 |  |
| Median Age  (range) | 61 (46-69) | 60.5 (41-68) | 62-(34-79) |  |  |
| Gender |  |  |  |  |  |
| Female | 2 | 5 | 4 | 11 |  |
| Male | 1 | 1 | 3 | 5 |  |
| Ethnicity |  |  |  |  |  |
| White | 3 | 5 | 6 | 14 |  |
| Asian (Indian/Bangladesh/Pakistani) |  | 1 | 1 | 2 |  |
| Performance Status |  |  |  |  |  |
| 0 | 1 | 3 | 3 | 7 |  |
| 1 | 2 | 3 | 3 | 8 |  |
| 2 | 0 | 0 | 1 | 1 |  |
| Prior lines of systemic therapy |  |  |  |  |  |
| 0 | 1 | 0 | 0 | 1 |  |
| 1 | 1 | 1 | 1 | 3 |  |
| 2 | 0 | 2 | 2 | 4 |  |
| 3 | 1 | 3 | 2 | 6 |  |
| 4 | 0 | 0 | 2 | 2 |  |

| **S2A Paclitaxel** |  | **Dose level 1**  **N (%)** | **Dose level 2**  **N (%)** | **Dose level 3**  **N (%)** | **All**  **N (%)** |
| --- | --- | --- | --- | --- | --- |
| **Reason for**  **stopping treatment** | **Patient completed protocol specified treatment** | 2 (66.7) | 2 (33.3) | 1 (14.3) | 5 (31.3) |
|  | **Patient progressed / died from disease** | 1 (33.3) | 3 (50.0)** | 2 (28.6) | 6 (37.5) |
|  | **Excessive toxicity (not including toxic death)** | 0 (0) | 0 (0) | 1 (14.3) | 1 (6.3) |
|  | **Other reason related to treatment** | 0 (0) | 0 (0) | 2 (28.6) | 2 (12.5) |
|  | **Other reason not related to treatment** | 0 (0) | 1 (16.7) | 1 (14.3) | 2 (12.5) |
| **S2B: Taladegib** |  | Dose level 1  (N) | Dose level 2  (N) | Dose level 3  (N) | All  (N) |
|  | **Reasons for delays***** | Other (2) | N/A | Other (2) | Other (4) |
|  | **Reasons for omissions**** | Patient error (3)  Other (1) | Patient error (3),  Toxicity (3)  Patient choice (3)  Other (2) | Patient error (1),  Toxicity (5)  Patient choice (2)  Other (2) | Patient error (7),  Toxicity (8),  Patient choice (5)  Other (5) |
|  | **Reasons for reductions**** | Peripheral neuropathy (2)  Other (1) | Peripheral neuropathy (3)  Neutropenia (2)  Other (2) | Peripheral neuropathy (1),  Neutropenia (3)  Other (2) | Peripheral neuropathy (6)  Neutropenia (5)  Other (5) |
